# The Patent Ductus Arteriosus in Adults with Special Focus on Role of CT

**DOI:** 10.3390/diagnostics11122394

**Published:** 2021-12-19

**Authors:** Soo Jeong Lee, Seung Min Yoo, Min Ji Son, Charles S. White

**Affiliations:** 1 Department of Radiology, CHA University Kangnam Medical Ceneter, Seoul 06135, Korea; 2jihyesung@chamc.co.kr; 2 Department of Radiology, CHA University Bundang Medical Ceneter, Seongnam 13497, Korea; smj3006@chamc.co.kr; 3 Department of Radiology, University of Maryland, Baltimore, MD 21201, USA

**Keywords:** computed tomography, patent ductus arteriosus, CT

## Abstract

The primary imaging modality for the diagnosis of patent ductus arteriosus (PDA) is echocardiography. However, CT may be the technique on which an incidental PDA is first recognized because of the increasing number of chest CT scans performed for a variety of causes. Identification of PDA on CT may lead to earlier closure using a PDA occluder device. Immediate identification of incidental PDA is important, but a high rate of missed diagnosis of PDA has been reported due to its small size and anatomic location. In addition, echocardiography may overlook the presence of even a large PDA due to decrease in the amount of shunting through the PDA caused by high pulmonary artery pressures. This review provides the basic CT anatomy and clinical perspective of PDA, and discusses the role of CT in the evaluation of PDA as well as methods to avoid overlooking a small PDA on CT.

## 1. Introduction

Patent ductus arteriosus (PDA) comprises approximately 10% of congenital heart disease. Most cases of PDAs are sporadic, although prematurity, viral infections such as rubella, and congenital heart disease are associated with PDA. The overall prevalence of PDA is about 1/500–1/2000 births [1,2,3]. The primary imaging modality for diagnosing PDA is echocardiography supplemented by various imaging modalities such as chest X-ray, cardiac catheterization, MRI, and CT [1,2,4,5]. However, CT may be the initial technique on which a PDA is identified because of the increasing number of chest CT scans that are being performed [6]. Therefore, it is imperative for interpreters to have an understanding of the diagnostic pitfalls of PDA on CT. This review provides a basic review of the CT anatomy of PDA, and discusses the potential role of CT in the diagnosis of PDA as well as methods to avoid overlooking a small PDA on CT.

## 2. Basic Embryology of PDA

The ductus arteriosus measuring about 5–10 mm connects the left main pulmonary artery to the descending thoracic aorta just distal to left subclavian artery during fetal life. At this time, the diameter of the PDA is similar to that of the descending thoracic aorta, although there is wide variation in its length. The main function of the ductus arteriosus during fetal life is to deliver unoxygenated blood from the superior vena cava to the descending thoracic aorta, and eventually to the maternal bloodstream via the umbilical artery and placenta, while bypassing the pulmonary circulation [7]. The patency of the ductus arteriosus during fetal life is maintained by low the partial pressure of oxygen within the PDA, high concentration of vasodilating local or circulating prostaglandin, and high concentration of local nitric oxide. After birth, abrupt changes including increased pulmonary venous return due to onset of respiration (i.e., increase in the partial pressure of oxygen), increased metabolism of prostaglandin by lung, and removal of the placenta trigger contraction of smooth muscle within the ductus arteriosus, resulting in occlusion of the ductus arteriosus and formation a ligamentum arteriosum [1,4]. However, if the ductus arteriosus is not obliterated within 48 h after birth, it is regarded as persistent, forming PDA.

## 3. Clinical Features of PDA

### 3.1. Small PDA

The clinical presentation of PDA is variable, ranging from congestive heart failure to completely asymptomatic mainly depending on the degree of shunt flow (i.e., the size of PDA and pressure difference between the aorta and pulmonary artery). Most patients with small PDAs are identified incidentally on physical examination (i.e., by a murmur on auscultation), echocardiography or CT performed for unrelated reasons, connoting a silent PDA in this case [1,4]. Although rare, symptoms of sepsis such as fever, hypotension, or bacteremia may occur in the patients with endarteritis.

### 3.2. Moderate to Large PDA

In most children with a moderate size of PDA the anomaly may remain silent until hypertension develops in adulthood. As hypertension may increase left-to-right shunt flow, such patients may present with symptoms of heart failure including dyspnea or exercise intolerance. A continuous machinery murmur along the left upper sternal border may be audible in these patients. A large-size PDA may be complicated by Eisenmenger syndrome due to severe pulmonary hypertension. In this situation, oxygenated blood from aortic arch proximal to PDA supplies the upper extremities. However, as unoxygenated blood is preferentially delivered to the lower extremity through the large PDA, isolated cyanosis in the lower extremities may occur [1,4].

## 4. Chest Radiographic and Echocardiographic Findings of PDA

Chest radiographic findings depend mainly on the degree of shunt flow. In patients with a small PDA, the chest radiograph is often normal. In contrast, enlargement of the left ventricle and main pulmonary artery is often identified in patients with a moderate size PDA or a small PDA associated with hypertension [1]. Echocardiography is the mainstay in the diagnosis of PDA. PDA and secondary findings caused by PDA (i.e., pulmonary hypertension, left atrial enlargement, and left ventricular enlargement and/or hypertrophy) can routinely be evaluated by echocardiography. Color Doppler echocardiography is most sensitive for identifying even tiny a PDA. Importantly, combined congenital abnormalities such as patent foramen ovale, aortic coarctation, and right sided aortic arch can also be identified on echocardiography. Moreover, the ratio in the diameter of aortic root to left atrium can be measured on echocardiography. If the ratio is more than 1.2, significant shunt flow may be present, suggesting the need for trans-catheter closure of the PDA. However, evaluation of PDA on echocardiography may be limited in patients with poor acoustic windows [1,2]. 

## 5. CT Anatomy of PDA

**Figure 1:**
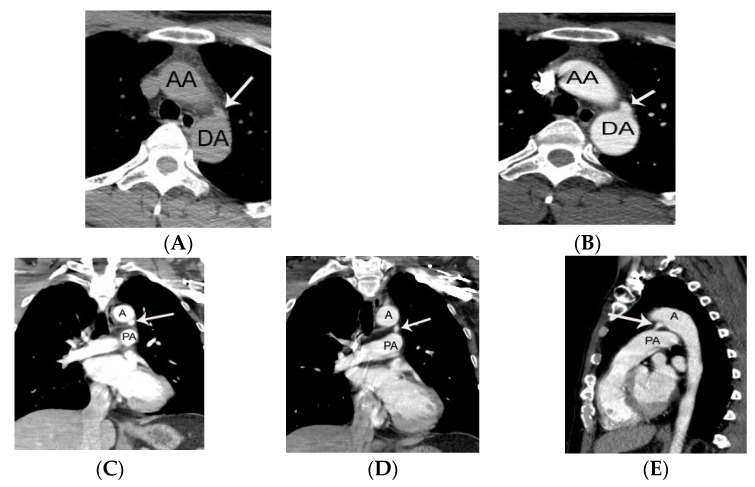
Representative case of small PDA initially diagnosed on non-gated chest CT in a 36-year-old male patient. A conically shaped protrusion (arrow on **A**,**B**) of PDA arising from the descending thoracic aorta at the 12 o’clock position is noted at the level of trachea on an axial CT image. The absence of calcification argues against a calcified ligamentum arteriosum. Note that the PDA is more easily identified on coronal (arrow on **C**,**D**) and sagittal CT images (arrow on **E**). AA, DA, A, and PA indicate ascending aorta, descending aorta, aorta, and pulmonary artery, respectively.

PDA is demonstrated on CT as an enhancing structure connecting the left main pulmonary artery to the descending thoracic aorta. It is helpful for radiologists to remember that on axial CT images the PDA is located near the twelve o’clock position relative to the descending thoracic aorta just distal to left subclavian artery (Figure 1).

**Figure 2:**
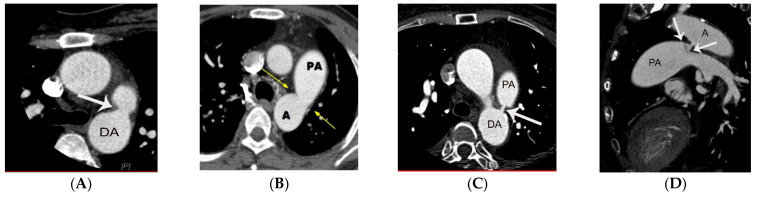
Representative cases of PDA subtypes (arrow on types **A**–**D**). ECG-gated curved multi-planar CT image (**D**) shows a peculiar Y-shaped PDA that has two openings at the pulmonic end of PDA. The case may be categorized as a type D or an unclassified subtype of PDA. DA, A, and PA indicate descending aorta, aorta, and pulmonary artery, respectively.

One study based on angiography has defined five subtypes of PDA [8]. Type A, the most common subtype is a conically shaped PDA. The conically shaped PDA has a larger diameter on its aortic side compared to the pulmonic end of the PDA. Type B and Type C PDAs have configurations that simulate a window (i.e., wide and short PDA) and tubular shape (i.e., similar diameter at the aortic and pulmonic end), respectively. Type D and E are rare subtypes defined by their complex and elongated appearances. Type D PDA may have multiple constrictions or a complex shape. An elongated PDA has a similar configuration to type A, but with a much more elongated appearance, and is often identified in infants. The subtypes of PDA of the angiographic classification can be used analogously on chest CT (Figure 2) [9].

**Figure 3:**
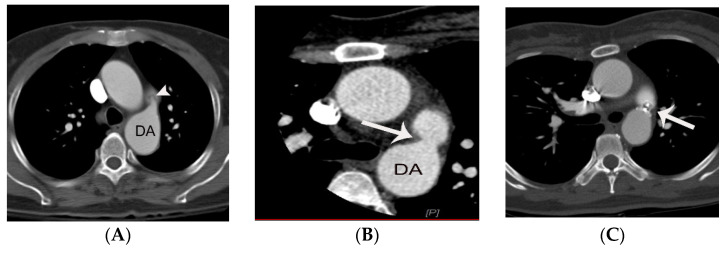
Representative cases of conically shaped PDA with a valve-like structure at the pulmonic end (arrow head on **A**) and in its absence (arrow on **B**). Amplatzer occluder device (arrow on **C**) was successfully administered in a patient with conically shaped PDA (Same patient as **B**).

The main strength of CT, especially ECG-gated cardiac CT compared to echocardiography is its ability to provide anatomic details (i.e., planimetry of PDA such as length, minimal diameter, and subtype) as a roadmap prior to closure with a PDA occluder device [10,11,12]. A recent study indicated a high prevalence of a linear valve-like structure at the pulmonic end of PDAs, especially the conically shaped PDA subtype, possibly due to the effects of chronic shear stress (Figure 3) [13]. Notably, the study included 38 patients with PDA in whom ECG-gated cardiac CT angiography was performed, leading to first identification of the valve-like structure at the pulmonic end. Enrolled patients were divided into group 1 (n = 16, patients with a linear valve-like structure at the pulmonic end) and group 2 (n = 22, those lacking a valve-like structure at the pulmonic end). The authors found that the valve-like structure was only identified in the conically shaped type PDA (*p*  =  0.04). In contrast, there was variability in the type of PDA in group 2. The geometry of a conically shaped PDA (i.e., larger entry diameter at the aortic end with smaller outflow tract) may induce stronger turbulent flow at the pulmonic end of PDA compared to other types of PDA. This is a possible explanation for why the valve-like structure was identified only in the conically shaped PDA [13]. Morgan-Hughes et al. evaluated 6 patients with PDA in whom ECG-gated cardiac CT was performed. However, the valve-like structure at the pulmonic end of PDA was not mentioned in this study. This valve-like structure may have not been identified in their study due to its subtlety and the small number of the patients [6]. The valve-like structure at the pulmonic end should be not misinterpreted as evidence of endarteritis on CT. However, a nodular thickening rather than a linear valve-like appearance may favor endarteritis [13,14]. Thus, a diagnosis of endarteritis should not be made merely by the presence of a linear valve-like structure at the pulmonic end of PDA in the absence correlative clinical information.

The degree of shunt flow in patients with PDA is determined by anatomic features of PDA (i.e., smallest diameter, length, and elasticity) and the pressure gradient between the aorta and pulmonary artery [1]. Overall shunt flow through the PDA is typically decreased in PDAs with smaller diameter, longer length, higher stiffness, and a lower pressure gradient between the aorta and pulmonary artery [1]. 

## 6. Potential Role of CT in the Diagnosis of PDA

### 6.1. Potential Role of CT in Moderate-to Large-Sized PDA

**Figure 4:**
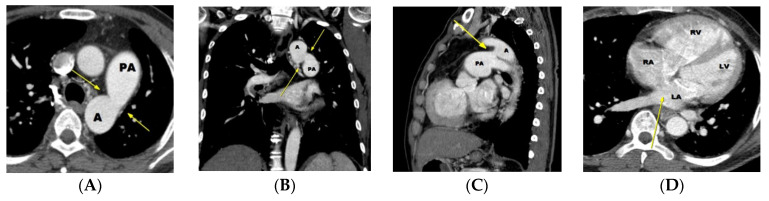
A case of PDA with Eisenmenger syndrome initially diagnosed by non-ECG gated chest CT. Moderate to large sized PDA (window shaped type **B**) (arrows) is noted on an axial (**A**), coronal (**B**), and sagittal (**C**) CT image. A and PA indicate aorta and pulmonary artery, respectively. A possible discontinuity (not atrial septal defect, but patent foramen ovale) is noted in the inter-atrial septum, but is not definite in the absence of ECG-gating (arrow on **D**). Note enlargement of the right atrium and ventricle due to Eisenmenger syndrome. Prior transthoracic echocardiography (obtained on seven occasions) failed to diagnosis the PDA over a period of 15 years, resulting in delay in diagnosis and obviating the opportunity for surgical correction. On echocardiography, the increased pulmonary artery pressure was falsely assumed to be due to an atrial septal defect. In fact, a large atrial septal defect is typically not an associated abnormality in patients with a large PDA because the presence of large atrial septal defect would trigger closure of the PDA. This case demonstrates the value of CT identification of PDA in some cases of a large PDA. LA, LV, RA, and RV indicate left atrium, left ventricle, right atrium, and right ventricle, respectively.

Most large-sized PDAs present with congestive heart failure at an early stage of life (infancy to early childhood) due to left heart volume overload. Diagnosis in most cases of large diameter PDA is straightforward on transthoracic echocardiography. However, a moderate to large PDA with significant increase in the pulmonary artery pressure (i.e., impending Eisenmenger status or Eisenmenger syndrome) may lead to decrease in flow through the PDA caused by a high pulmonary artery pressure [1]. In this situation, a PDA may be overlooked on echocardiography due to scanty shunt flow (Figure 4).

In contrast to echocardiography, CT can visualize the PDA itself, irrespective of the amount of shunt flow. Thus, CT identification of a moderate-to large-sized PDA before developing Eisenmenger syndrome is critical because it may provide a chance for surgical or interventional treatment.

### 6.2. Potential Role of CT in Small PDAs

**Figure 5:**
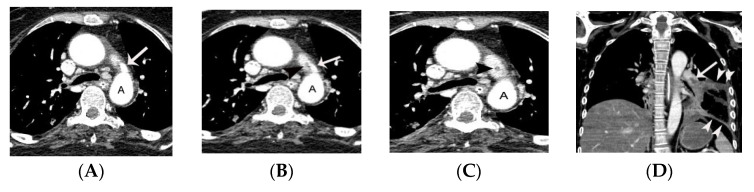
Septic pulmonary infarction caused by embolization of vegetation or endarteritis of a PDA in a 42-year-old female with history of glioblastoma multiforme. Small conically shaped PDA (arrow) is noted on contiguous axial CT images (**A**–**C**). Note small nodular lesion (arrowhead on **C**) at the pulmonic end of the PDA, suggesting a vegetation or endarteritis. This finding was confirmed by subsequent echocardiography. Septic embolus is noted in the pulmonary artery in the left lower lobe (arrow on **D**) on a coronal CT image at the level of descending thoracic aorta. Septic pulmonary infarction with necrosis (arrowheads on **D**) is also demonstrated in the left lower lobe. This patient eventually died due to septic shock. A indicates aorta.

The risk of arterial infection endarteritis in patients with PDA has decreased because of improved dental and health care, widespread use of broad-spectrum antibiotics, and increased frequency of PDA closure using an interventional occluder device [1]. However, endarteritis can still occur, particularly with a small or silent PDA [Figure 5] [14,15,16,17,18,19]. Turbulent flow secondary to abrupt change in diameter and pressure through the small PDA may lead to formation of endarteritis at the pulmonic end of PDA. The lung, specifically pulmonary septic embolism secondary to PDA endarteritis may be the primary target in the embolic events (Figure 5C,D) [2]. Table 1 summarizes the pros and cons of CT in the evaluation of PDA compared to echocardiography and MRI. 

Earlier detection of small size PDA is important because it may lead to earlier intervention (i.e., percutaneous closure of PDA with occluder device). Despite the importance of identification of incidental PDA on CT, a high false negative diagnosis rate has been reported for silent PDAs study [9]. In a recent study, the diagnostic accuracy of silent PDA on CT was poor. The authors retrospectively analyzed non-ECG gated chest CT in 64 patients with PDA. These patients were divided into group 1, those who had pretest information and group 2 who lacked pretest information. Chest CTs were read by eleven radiologists. The authors assessed whether a PDA was described on the initial CT reading. The diagnosis of PDA was made in all the patients in group 1 (100%, 42/42), whereas a correct diagnosis was made in only 13.7% in group 2 (3/22) on routine 3 mm chest CT. This result was attributed mainly to the small size of the PDAs and their anatomic location (i.e., a small structure between the aorta and main pulmonary artery demonstrating strong enhancement). To avoid a false negative diagnosis, the authors recommended analysis of axial CT images with thinner slice thickness (1–2 mm) and the use of multiplanar reformatted images such as coronal and sagittal views for better evaluation of small PDAs [9].

## 7. Diagnostic Pitfalls of PDA on Chest CT

**Figure 6:**
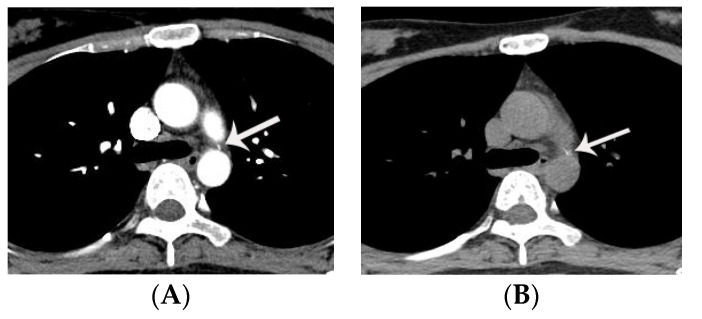
Typical case of calcified ligamentum arteriosum in a 32-year-old female patient. Calcification within the ligamentum arteriosum may be confused with a small PDA if only enhanced chest CT is obtained. Note calcification (arrow on **A**,**B**) within the ligamentum arteriosum at the level of tracheal bifurcation.

Almost half of adults have calcification within the ligamentum arteriosum (Figure 6). Calcification within the ligamentum arteriosum may be confused with a small PDA on enhanced chest CT. Thus, interpreters suspecting a small PDA on enhanced CT should confirm that there is no evidence of calcification within the ligamentum arteriosum on non-enhanced CT.

**Figure 7:**
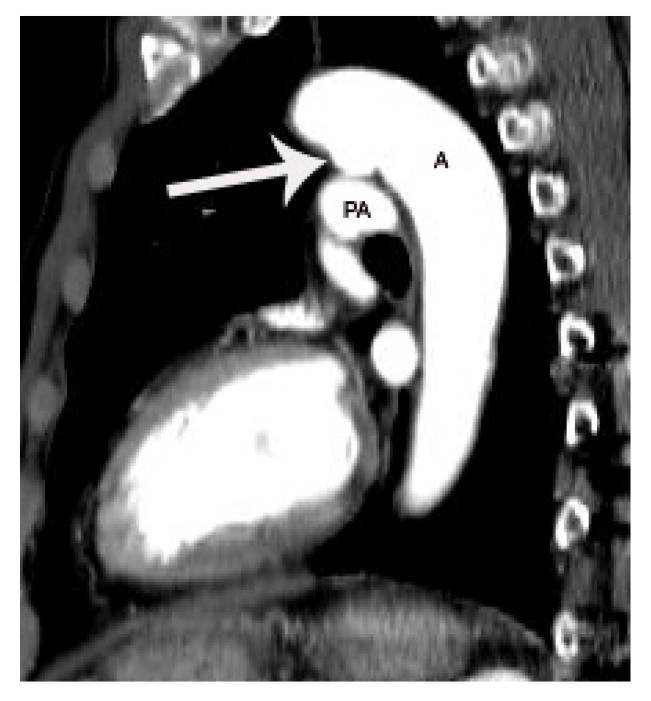
Representative case of a ductus diverticulum in a 60-year-old male, as demonstrated on a sagittal CT image at the level of aortic arch. Although a ductus diverticulum (arrow) may simulate the CT features of conically shaped PDA at the aortic end, it lacks a connection with the left main pulmonary artery.

In addition, a ductus diverticulum should not be confused with a conically shaped PDA. The CT appearance of ductus diverticulum is similar to that of conically shaped PDA. However, a connection to the left main pulmonary artery is absent in the former (Figure 7). Aneurysm of the ductus arteriosus has been reported more frequently in children than in adults. Aneurysm of the ductal diverticulum should not be confused with other mass lesions of the aorticopulmonary window [2].

## 8. Treatment of PDA; Surgery versus Intervention

### 8.1. Surgical Treatment

Surgical ligation or division is the primary option for PDA closure in a premature infant (<2 kg) with failed optimal medical treatment or in a patient with a very large PDA or unsuitable anatomy such as aneurysm formation [1,20,21]. This surgery is performed using a left posterolateral thoracotomy approach without cardiopulmonary bypass. Complication of surgical ligation includes bleeding, recurrent laryngeal nerve palsy, infection, chylothorax, and pneumothorax. However, technical advancements today permit even some large PDAs to be treated by percutaneous closure [22,23] with a lower morbidity rate and lesser postprocedural pain compared to surgery. 

### 8.2. Transcatheter Closure of PDA

#### 8.2.1. Ideal Device for Transcatheter Closure of PDA

Portsmann et al. performed the first trancatheter closure of PDA in 1966. This was done using an Ivalon plug with an 18 F catheter [24]. Since then, there have been remarkable advances in the technique for transcatheter closure of PDAs [24,25,26,27,28,29,30,31,32,33,34,35], and evolution is continuing with the following aims for the closure device: (1) making the device with material compatible with MRI; (2) conforming it to the full spectrum of PDA sizes and shapes; (3) permitting it to be safely delivered with a sheath of small diameter; (4) allowing free retrievability of the device; (5) crafting a device that will permanently occlude the PDA. Closure by an interventional occluder device is the primary therapeutic choice in infants, children, and adults with a small or moderate size symptomatic PDA [1,11,23,24,25,26,27,28,29,30,31,32,33,34].

#### 8.2.2. Potential Use of CT Prior to Transcatheter Closure of PDA

Coronary angiography has been used as the standard method of planimetry to assess the PDA [23]. However, angiography has limitations in evaluating the precise minimal and maximal diameter and length of PDA because of the two-dimensional nature of angiography. Improper measurement of the minimal diameter or length of PDA may be associated with serious complications such as embolization of the device [24,25]. In contrast to angiography, CT, especially ECG-gated cardiac CT angiography can be used as a road-map by precisely providing anatomical details and planimetry of the PDA (e.g., length, minimal and maximal diameter of PDA) prior to intervention by using the three-dimensional multi-planar reformatted images such as curved multi-planar image and volume rendering [13]. 

#### 8.2.3. Which Is the Best Option for Transcatheter Closure of PDA 

There are four types of devices now utilized in the USA (i.e., Gianturco coil, Flipper coil, Amplatzer duct occluder, and Nit-Occlud) [25]. In general, the first two devices are used in PDA patients with a minimal diameter < 2 mm. In contrast, the Amplatzer duct occluder and Nit-Occlud are best employed in PDA patients with a minimal diameter > 2 mm and sufficient size of the aortic ampulla [24,25]. These last two devices are associated with a higher success rate and a lower complication rate as compared to the first two [25]. The specific type of device should be chosen based on the shape of the PDA (i.e., subtype of PDA) and minimal diameter and length of the PDA. 

#### 8.2.4. Complications of Transcatheter Closure of PDA

Incomplete closure of PDA is rare, but important complications related to incomplete transcatheter closure of PDA are endarteritis or hemolysis secondary to a high velocity shunt flow [24]. This complication is more frequently identified with the use of Gianturco or Flipper coils. It should also be remembered that delayed occlusion of the PDA after application of Amplatzer occluder device can occur. Thus, completion of transcatheter closure of PDA is feasible if the device is located in the proper position. In contrast, transcatheter closure of PDA may not be completed in cases where coils are used, and complete occlusion of PDA should be confirmed on angiography. Incomplete closure should be treated by application of a second device. 

Embolization of the PDA closure device to the pulmonary artery or aorta may occur. This complication can be treated by catheter-based intervention or surgery. Therefore, some experts recommend that transcatheter closure of PDA be done only in hospitals where immediate surgical rescue is possible. Stenosis in the pulmonary artery and descending thoracic aorta is a rare complication of transcatheter closure of PDA. This complication commonly occurs in patients with small body weight and a relatively large size PDA [24]. In addition, peripheral vessel injury may occur. 

#### 8.2.5. Controversy of Transcatheter Closure in Patients with Small Silent PDA

**Figure 8:**
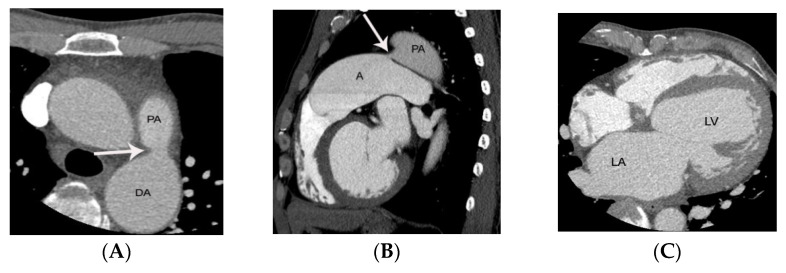
PDA diagnosed initially by ECG-gated cardiac CT, leading to closure with Amplatzer duct occluder in a 46-year-old male patient who presented with dyspnea. Conically shaped PDA without valve-like structure at the pulmonic end is noted on axial (arrow on **A**) and sagittal (arrow on **B**) CT images. Left atrial and ventricular enlargement is demonstrated on an axial CT image at the level of the left ventricle (**C**). PDA closure by Amplatzer duct occluder was performed in this patient due to left atrial enlargement (dyspnea). ECG-gated cardiac CT angiography provided excellent anatomic detail and planimetry without motion artifact prior to PDA closure. DA, PA, LA, and LV indicate descending thoracic aorta, pulmonary artery, left atrium, and left ventricle, respectively.

Another important issue is whether silent PDAs should be treated by interventional occluder insertion. As neither the risk for endarteritis and complications of interventional procedure are high, controversy exists. Interventional occluder insertion may not be recommended for all silent PDAs based on economic considerations. Further randomized controlled study may address this issue. Thus, interventional occluder insertion is often restricted in patients with silent PDA to those with enlargement of the left atrium due to the risk of left heart failure (Figure 8) [1].

#### 8.2.6. Transcatheter Closure of PDA in Patients with Severe Pulmonary Hypertension

The decision on whether to perform transcatheter closure of PDA in patients with severe pulmonary hypertension is challenging. In general, severe pulmonary hypertension is defined by pulmonary pressure that is more than two-thirds of systemic pressure [1]. In such patients, morphologic changes may occur in the peripheral pulmonary arterioles (i.e., arteriolar medial hypertrophy, intimal proliferation) leading to increase in the pulmonary vascular resistance. The decision regarding whether to occlude the duct in these patients should be based on the pulmonary response to vasoreactivity testing (i.e., inhaled nitric oxide) or lung biopsy [24].

## 9. Conclusions

Precise understanding regarding the pros and cons of the various diagnostic modalities may lead to prompt diagnosis of PDA. A timely diagnosis of PDA may result in better outcome.

## Figures and Tables

**Table 1:** Pros and cons of the echocardiography, CT, and MRI in the evaluation of PDA.

	Echocardiography	CT	MRI
Functional evaluation	++	−	+
Diagnosis of PDA with severe pulmonary hypertension	+/−	+	+/−
Diagnosis of silent PDA	+	+	−
Radiation hazard	−	+	−
Iodine hypersensitivity	−	+	−
PDA indicates patent ductus arteriosus			

## Data Availability

Not applicable.
